# Haunted Aspirations and Youth Transitions in the Contemporary Context

**DOI:** 10.1007/s43151-025-00161-6

**Published:** 2025-02-13

**Authors:** Charlotte McPherson

**Affiliations:** Youth Research Collective, Faculty of Education, University of Melbourne, 100 Leicester Street, Parkville, VIC 3052 Australia

**Keywords:** Youth, Temporality, Transitions, Hauntology, Social change

## Abstract

Contemporary generations of youth are undertaking their “transitions” to adulthood in a period marked by considerable temporal precarity and anxiety. A range of intersecting socio-structural changes and crises have not only made it more difficult for contemporary young people to achieve historically conventional markers of adulthood but have also undermined the previously taken-for-granted sense of continuity between the present and future. Youth studies have been alert to how these shifts have reshaped young people’s lives in the present and their dispositions towards uncertain futures. Significantly less attention has been paid to how this temporal uncertainty evokes the past, present and future in young people’s own perceptions and navigations of the contemporary context. Using the concept of hauntology and drawing on the narratives of 11 working-class young people as they reflect on their transitions to adulthood, this paper provides insights into how young people’s experiences and dispositions in the present are powerfully haunted by losses from both the past and the future.

## Introduction

Youth studies scholars have strongly critiqued the spectre of futurism that encircles youth and young people (e.g. Goring et al. 2023; McPherson 2024), pointing out that it minimises the importance of young people’s lives in the present and overlooks the significance of the past by relying upon the assumption of a “flat linearity” in the temporal cadence of young people’s lives and transitions (McLeod and Yates 2006: 31). Despite these critiques, youth studies research, too, often places the temporal emphasis of youth on the future. This is evident in the dominance of youth transitions research and, more recently, in relational and temporal “turns” which have valuably diversified the field but have maintained an overwhelming focus on the future by examining young people’s life trajectories and “future imaginaries” in the context of social change and crisis (Wood 2017; McPherson 2024). While the future is undoubtedly an important concern for youth researchers, the disproportionate emphasis on the future that surrounds youth has contributed to problematic understandings of the present and future as temporally disembodied from the past which, in turn, has drawn attention away from the past and its significance for young people’s lives, including their transitions to adulthood and subjective orientations to the future.

A number of scholars have noted the curious absence of socio-historical analysis from much youth studies research and have proposed a range of ways to incorporate it into the discipline, including adopting genealogical rather than linear conceptions of time (e.g. Wood 2017; Feldman-Barrett 2018). In a recent article, I suggested that Derrida’s (1994) concept of hauntology represents a timely and valuable conceptual lens through which to engage with central research concerns in youth studies in a way that recognises and explores the significance of temporal interconnections between the past, present and future (McPherson 2024). In this article, I use the concept of hauntology to analyse how a group of Scottish young people’s narratives about their lives and transitions to adulthood appeared to be powerfully haunted by “ghosts” from the past and future. The article begins with a critical discussion of youth and temporality, which is followed by a consideration of the concept of hauntology and a description of the research study. The data is then analysed over three sections, which explore how the participants’ narratives about their transitions to adulthood were haunted by intergenerational disparities, the ghosts of class and place, and a sense of injustice.

## The Temporal Framing of Youth

Young people’s emergent position in the life-course means that they are often viewed and used as proxies for the future—as figures of hope, anxiety and risk (Threadgold 2020). Youth scholars have been critical of the futurism that surrounds young people, arguing that it, among other things, overlooks the significance of young people’s lives in the present, contributes to neoliberal constructions of young people as economic subjects, and operates on the assumption of linearity in young people’s trajectories towards adulthood (e.g. Cuervo and Wyn 2014; Harris 2015; Cuervo and McPherson 2024). However, scholars have also noted that youth studies itself is dominated by a focus on the future (Wood 2017; Feldman-Barrett 2018; McPherson 2024). This is most clearly evident in the dominance of youth transition research, which can share parallels with the emphasis on transition in youth policy in its exploration of young people’s movements between education and employment and their implications for both societies and young people in the future. While it remains dominant, transitions research has been strongly critiqued over the last two decades, partly due to the futurism inherent in its approach, but also due to the impact of significant social change and a corresponding de-standardisation of the life-course (Pimlott-Wilson 2017). This has led to the emergence of a “new transitions framework” within youth studies, which considers how socio-structural forces like globalisation, neoliberal capitalism, temporal uncertainty and risk have radically changed young people’s life trajectories (Harris 2015: 86).

It has also led to a growing interest in temporality in youth studies, reflected in sociology more broadly, in recognition that the contemporary period is marked by disorientating and intense social change (Woodman 2011). The intensity and pace of this change hase not only destabilised life trajectories but is believed to have generated a suite of temporal anxieties in the twenty-first century, where the previously taken-for-granted sense of continuity between the present and the future has been undermined, infusing the contemporary context with uncertainty and casting the security of the future into doubt (Fuchs and Detmers 2017). Leccardi (2015: 42) argues that this uncertainty has erased the “notion of the life-plan as developed in the modern era”, which is particularly significant for young people, given that the purpose of youth as a life-stage has been so powerfully and enduringly delineated as preparation for adulthood (McPherson 2024). A growing body of research within youth studies now examines how young people navigate these uncertainties in the present and the increasing difficulties contemporary generations are facing in meaningfully preparing for their futures amid weakened opportunity structures, low levels of state support, rising living costs and the existential threats posed by climate change (e.g. Bone 2019; Walsh and Gleeson 2022; Goring et al. 2023).

In many studies, this has involved adopting a relational focus. Cuervo and Wyn (2014) advocate understanding youth from a relational perspective through the metaphor of belonging; an approach that considers how young people work to belong in the conditions of late modernity. One register of belonging they focus on is young people’s relationships with place. A plethora of research in youth studies and beyond has highlighted the significance of place for young people in contemporary society, where it has been found to represent a strong source of belonging, a key mechanism in the cultivation and maintenance of identity and social connections, and an anchor of familiarity and rootedness amid considerable temporal and structural uncertainty (e.g. Riethmuller et al. 2021; Areschoug 2024; Cuervo and McPherson 2024). This growing temporal and relational focus in youth studies has, in some ways, shifted the temporal emphasis in youth studies from the future to the present, the temporal time zone where young people are increasingly perceived to be “stuck”. However, much of this research retains a futural emphasis, with a growing number of studies interested in young people’s “imagined futures” in the context of risk, insecurity and crisis (e.g. Johnson and West 2022; Bazzani 2023).

Contemporary research in this vein has been instrumental in generating important insights about how differently positioned young people work to cope and build lives in a challenging socio-structural and temporal context. However, as I argued recently (McPherson 2024), there has been a significant lack of recognition of the interplay between the past, present and future, and how this temporal confluence can be key in shaping young people’s experiences, perceptions and orientations in the contemporary context. A small group of scholars have sought to disrupt the deference to linear time and the future in traditional youth transitions research and policy by illustrating that as young people progress towards or think about the future, they do so inevitably carrying the weight of their history (e.g. Dillabough and Kennelly 2010; Wood 2017). They suggest that the fixation in youth studies on the present can only ever cultivate incomplete and decontextualised snapshots of young people, overlooking the significance of their “sedimented past” and its critical role in their experiences and perceptions in the present. Wood (2017) and others (McLeod and Yates 2006; Hall et al. 2009) have also been critical of the overwhelming attention paid to social change in contemporary youth studies, arguing that such change coexists and is embedded within continuities for young people—continuities which can play a significant role in how/if this change is perceived and navigated.

The precarity and uncertainties of the contemporary period have demanded a rethink of young people’s transitions. It has also called into question the grand narratives that have long underpinned these transitions, including modernity’s promise of improvements in the future and, perhaps more fundamentally, the transformation of the present from a moment of transition to a timeless limbo for young people (Fuchs and Detmers 2017). While youth studies research has excelled at demonstrating the impacts of social change on young people’s economic transitions, there is a need for more research that transcends either a disembodied emphasis on the future or that submits to “amnesia of the now” in its scrutiny of the present (Wood 2017). In the following section, I discuss Derrida’s (1994) concept of hauntology, the *pantemporal* approach of which offers a valuable lens through which to better analyse and contextualise the experiences of contemporary youth and transitions.

## Hauntology

Hauntology was first theorised by Derrida (1994) in his critique of neoconservative interpretations of the collapse of the Soviet Union as signalling the “death” of socialism with the rise of capitalism. Derrida (1994: 46) rejected this position, arguing that, far from being dead, “Marx’s ghosts” lingered in the air and haunted capitalist Europe with thoughts of what could have been. Despite Derrida’s focus on the specific example of the collapse of the Soviet Union, he believed haunting to be an inescapable part of human and societal life, and that hauntology should be understood as a theory of everyday life (Rahimi 2021). A hauntological approach involves recognising that the present is “scarcely as self-sufficient as it claims to be”, and in fact is affectively encroached upon by both the past and future, particularly in moments of change, danger and difficulty (Jameson 1991: 39; Lincoln and Lincoln 2015). These “historical [whispers]” saturate the present with an eerie atmospheric excess that colours social relations with the sense that a part of history and, in turn, an alternative future, has slipped away (Ahlberg et al 2021: 165).

Hauntology resurfaced in the twenty-first century in the work of Mark Fisher (2009; 2014), who interpreted and applied the concept through a darker and more dystopian lens. Indeed, while Derrida focused on the haunting spectre of socialism amid triumphalist proclamations about the utopic potential of capitalism, Fisher’s hauntology centres on the haunting quality of the *loss* of this optimism in the future (Reader 2021; McPherson 2024). Fisher (2009) argues that as neoliberal capitalism has advanced and become increasingly harmful and inescapable, modernity’s promises of continuous progress and better futures have evaporated, creating an affective atmosphere of creeping dread about the “slow cancellation” of the future. In this affective atmosphere, Fisher (2009: 27) argues that people are no longer able to think—let alone move—beyond the present, so haunted are they by the unstoppable “failure” of the future and the chasm that exists between this and the futures they have been conditioned to expect.

Both Derrida and Fisher understood hauntology in relation to a crisis of time, “where past and future are present, and present is absent”, and where the ghost functions as “an advocate of the promised future that was unrightfully cancelled when the past was destroyed” (Rahimi 2021: 6). Haunting has two key functions: to signal that something has gone awry; and to act as a form of resistance to forgetting or overlooking this (Gordon 2008). Haunting, for Derrida (1994: 46), “belongs to the structure of every hegemony” that seeks to suppress and silence previous ways of life and alternative possibilities. As Avery Gordon (2008: xvi) puts it, “The appearance of spectres or ghosts is one way… we are notified that what’s been concealed is very much alive and present”. Accordingly, hauntology can be understood as a resistant and revolutionary concept that requires “a politics of memory, of inheritance, and of generations” that is critical to the pursuit of social justice (Derrida 1994: xviii).

The haunted atmospheres described by both Derrida and Fisher have only intensified in the years since. The creeping sense of dread about lost, failed and cancelled futures has a growing sense of resonance in the contemporary context, where the climate crisis, spiralling living costs and inequalities, political instability and war are gravely undermining a sense of security in both the present and the future (see, for example, Kishik and Pors 2024). The applicability of these concerns and conditions to contemporary generations of youth is striking, yet there has been a surprising lack of hauntological research with young people (McPherson 2024).

## Methods

This paper draws on a subset of qualitative data from a study investigating working-class young people’s experiences and perceptions of social justice in relation to their transitions to adulthood. Fieldwork for the study was carried out in 2019 and 2020 with a total of 42 young people (aged between 14 and 29) living in two different areas of central Scotland (a city and a small rural town). This paper focuses on the data collected with the 11 participants over the age of 18 who lived in the small town, which will be referred to using the pseudonym of Malden.

### Recruitment and Participants

Participants were primarily recruited to the study with the support of gatekeeping organisations (local employability initiatives, training organisations, further education colleges), who shared information about the research with young people. Several young people were also recruited to the study by word of mouth from existing participants. If a young person showed interested in taking part, they contacted the researcher, who contacted them shortly thereafter to confirm their eligibility and intent to take part, and to provide further information and the opportunity for them to ask any questions. Young people were provided with information sheets describing the research and consent forms.

The sampling criteria for the study stated that young people were eligible to take part if they were between the ages of 13 and 30 and if they lived in either “Gifford”, an urban city in Scotland’s central belt, or in “Malden”, a small ex-industrial town in the countryside of central Scotland. This study was specifically interested in the experiences of working-class young people. Sampling for social class is complex, in that it entails a combination of economic and social factors, as well as both more objective and subjective determinations. Sampling in this respect was therefore guided by a range of considerations. Participants were primarily recruited through programmes and services specifically targeted at socioeconomically disadvantaged young people and with the input of gatekeepers who had established relationships with the young people they worked with. In addition to this, prospective participants were asked a number of screening questions in efforts to approximate their social class background. The young people were asked to provide their postcode and neighbourhood of residence, along with information about their income, occupation and level of education. The same information was requested about their parents/carers. This information was then analysed in relation to social class using the NS-SEC classification (ONS 2024) and the Scottish Index of Multiple Deprivation (SIMD 2020).

Among the 11 young people over the age of 18 in Malden who took part in the research, 7 identified as male and 4 identified as female. The youngest participant was 19 and the oldest was 29. The sample reflected a range of statuses in relation to education and employment. Two participants were engaged in full-time further education and training at a vocational college. In terms of employment, of the 11 participants, six were in employment, while the remaining five were unemployed, including three categorised as “NEET”^1^ (not in education, employment or training). The participants’ names have been replaced with pseudonyms.

### Data Collection

Data was collected using semi-structured interviews that were conducted in person in private spaces within youth organisations or in public spaces in the local area (cafes and the library). During the interviews, participants were asked open-ended questions about their perceptions and experiences of opportunity structures and prospects in Malden, their aspirations and expectations for the future, and whether they felt they were experiencing fairness in relation to their transitions to adulthood. With consent from participants, all interviews were recorded and later transcribed verbatim. The interviews lasted between 30 and 50 min on average.

### Data Analysis

The interview transcripts were uploaded to NVivo, where they organised and labelled according to the young people’s ages, genders and education/employment status. The data were then subjected to thematic analysis. As described by Braun and Clarke (2006), this process consisted of several steps: (1) data familiarisation—the transcripts were read several times to provide an overview of the research findings; (2) generation of initial codes—using open coding, the transcripts were reviewed and anything that seemed relevant or interesting was coded; (3) searching for themes—the initial codes were reviewed and, where appropriate, consolidated into themes; (4) reviewing of themes—the coherence and validity of the themes were reviewed by attributing all relevant data from the transcripts to each theme and modifying themes as appropriate; (5) definition of themes—all identified themes were subjected to a final refinement, which consisted of generating a thematic map which explicated links between themes and between main themes and subthemes, providing a comprehensive picture of data linkages.

## Research Findings

The following sections present the three themes that were identified during data analysis in relation to haunting. The first details how the participants’ narratives about their aspirations seemed to be powerfully haunted by the past and anxieties about the future. In the second section, I discuss how ghosts of class and place were present in the young people’s reflections on their transitions to adulthood in the context of the deindustrialised town of Malden. Finally, in the third section, hauntology is used to provide insights into the participants’ sense of injustice in relation to their lives and futures.

## “I thought it would be the same”: Haunted Aspirations

Research in youth studies and beyond has long identified the significant influence of parents and families on the education and career dispositions, aspirations and decision-making of young people (e.g. Butler and Muir 2017; Lahelma and Gordon 2008; Wang et al. 2020). In this study, the participants’ aspirations and expectations for their lives and futures were highly influenced by the life trajectories of their older family members. Many participants had abandoned their original aspirations after encountering difficulties that made them feel unattainable and described looking to the routes taken by family members because they viewed them as “well-trodden paths” to employment undertaken by people they knew and shared the same socioeconomic background and geographic location with (Pimlott-Wilson 2017: 289). Pickering and Keightley (2006: 921) have argued that, while turning to the past is often derided as unproductive, it often represents an active attempt to “[take] one’s bearings for the road ahead in the uncertainties of the present”.

We’re a Malden family and nobody’s rich around here, but my parents got there in the end, they just plugged away at it… I’m not expecting to have a mansion or drive about in a BMW, but I do look at my mum and dad, the fact they’ve got their own wee house, they’ve got a car… It reassures me that I can surely go the same way. (Natalie, 20)

However, older participants were increasingly being confronted by a gap between their expectations and experiences. For example, while all the participants wanted to find full-time employment, only one out of the six participants in employment had a full-time job, while the rest were involuntarily employed on casual or part-time contracts in low skilled, low-paid jobs in retail and hospitality. While the participants had anticipated and accepted that these jobs would likely be their initiation into the labour market, they had also expected to transition into higher skilled, better paid, secure jobs once they had amassed work experience and/or further qualifications. As the young people moved through their twenties without experiencing meaningful changes in their circumstances or prospects, there seemed to be a growing recognition that these jobs were not the temporary “stepping stones” into secure work they had imagined (MacDonald 2009).

I’m very concerned about the future… just, nothing is going how I thought it would. My dad always said the thing that saved him from a life on benefits was getting a trade behind him, so I did that, and I thought it would be the same and here I am on and off benefits, scrapping for random bits of work like I’m still 18. (Steven, 25)

As the labour market has become progressively less secure for young people, youth studies research has captured a growing sense of frustration and concern among young workers who are struggling to access secure employment and to make other corresponding life transitions (e.g. Bone 2019; Cuervo and Chesters 2019; Nielsen et al. 2024). In the present study, the participants’ inability to access secure work left many of them feeling pessimistic about their capacity to leave home, start families and establish careers. This not only trapped them in the present, but also seemed to lead them backwards, to thoughts of how things apparently used to be for previous generations and why their own transitions to adulthood were unfolding so differently.

I just can’t stop thinking about how different it was for my parents… in terms of everything, just getting settled into life… The thing is, I have done everything they tell you to do… Fair enough, school was a write off, but I went to college, I did – I think it was three – courses, but there’s no jobs, so it’s hard to know what else you can realistically do to progress… I have no clue about the future anymore. (Leigh, 26)

The mismatch between the participants’ experiences and their aspirations/expectations can be understood as a haunting from both the past and the future in the ways Derrida (1994) and Fisher (2014) have theorised. The young people were haunted by both the foundering of historical youth transition patterns and by the visceral embodiment of this in the contrast between the biographies of their parents and their own experiences. The young people in this study were transitioning to adulthood in a contemporary atmosphere of increasing precarity, marked by the erosion of traversable paths from the present into the future and compounded by an unsettling lack of alternatives, charging the present with an unconscious sense that the future is being slowly cancelled (Fisher 2014; Ahlberg et al. 2021). In this context, the past can become “not only attractive, but the only open avenue” (Ahlberg et al 2021: 163), offering the young people a seemingly tangible example of a way forwards and/or functioning as a device for them to try and make sense of their own experiences in the contemporary present. In this sense, even in the highly futural act of considering their hopes and expectations for their futures, the participants encountered the past (Gammeltoft 2013) and in ways that sparked concern about their futures.

## Ghosts of Class and Place

There has been an increasing interest in young people’s relationships with place within youth studies in recent years. This is in part rooted in critiques of influential sociological arguments that suggest that, amid globalisation, (a) the significance of local places and identities has declined and, connectedly, that (b) mobility—and migration towards urban centres in particular—is not only required to make “successful” transitions to adulthood but has now displaced fixity as the norm (Cuervo and Wyn 2014; Stockdale and Haartsen 2018; Schewel 2019). Research in youth studies has challenged these positions, finding that many young people are emotionally attached to and socially embedded in their local areas; attachments that have been found to be key in young people’s identity construction, sense of belonging, and the decisions they make about their lives and transitions to adulthood (e.g. Farrugia 2016; Areschoug 2024; Cuervo and McPherson 2024). Dillabough and Kennelly (2010) argue that young people’s perspectives and actions should be understood intersubjectively as shaped by regulatory webs of social relations and historical narratives that are themselves strongly entwined with time, place and social class (Wood 2017). The strong emphasis in youth studies on social change and the future can lead to continuities in the lives, identities and communities of young people to be overlooked. Hall et al. (2009) note, for example, that even ruptures like deindustrialisation occur in tandem with continuities that shape young people’s experiences and orientations.

One popular application of hauntology has been in sociological analyses of the lingering effects of deindustrialisation on communities in the present. While the most acute period of deindustrialisation occurred in the 1980s, these studies have shown how legacies of industry and deindustrialisation can remain embodied in the present, haunting young people born over a decade after mass closures (Simpson 2021). For example, Geoff Bright’s (2011, 2012) research with working-class young people in former mining villages in England found that young people are profoundly (albeit often unconsciously) connected to and affected by local working-class culture and spatial biographies of deindustrialisation.

The participants in this study, who lived in the deindustrialised town of Malden, were similarly haunted in ways that entangled with their classed and spatialised identities. While the participants did not have direct memories of the town’s industrial history, most were highly aware of it, having grown up in working-class families and communities with deep connections to it, and they were equally aware of Malden’s subsequent slide into economic depression in their lifetimes. Several discussed being told about the town’s history at school and, more commonly, by family members, and expressed the opinion that the town had declined with deindustrialisation.

I don’t know that much but I know it was once a proper bustling place, full of work and had more life to it… I’ve heard it off my granddad and my aunty and uncle, but you can see it around the place… there’s just nothing going on. (Lisa, 21)

Research with young people has found that contemporary young men are often highly affected by deindustrialisation. In her recent study, Areschoug (2024) found that young men in rural Sweden expressed resistance to urbanisation, “newness” and difference, embodied in a proud defensiveness of local traditions and culture that strongly informed their identities and decision-making. The young men in the sample seemed particularly affected by what they perceived as Malden’s loss of identity, status and prosperity; a rupture in the town’s legacy that they experienced as an affront to their own identities as working-class young men in the town. Finlay, 27, was unemployed at the time of fieldwork but spoke passionately about the stark contrast he perceived between the jobs available in the local area now and in the town’s past.

I look at my dad and my granddad… and the sort of jobs and lives they had at my age and it’s laughably different to what we have in front of us today as young people in Malden today… They did proper work at the factories or the farms and that’s what the jobs were like here for families like mine… We seem to be living in a time where it’s okay to just force people to be cleaners or till monkeys or whatever… Where’s the skill? Where’s the respect in any of it?

Deindustrialisation has occurred at a relatively slow and disorientating tempo in Malden. While many manufacturing businesses closed during the 1980s, some remained open until later or had closed and reopened at various points in the years since. One factory, a major employer in the town for centuries, remains open, though now largely employs people via an agency and on insecure contracts. Its physical presence in the town, together with other physical remnants of Malden’s industrial past, meant that haunting seemed to be experienced by some participants in a particularly visceral way. Joe, 24, described how it felt to walk past the (much changed) factory in Malden.

I don’t know how to explain it properly but you’re walking past this big building that used to be like a big thing for Malden, it’s just… turn the corner and it’s the new Malden reality… the closed-up shops, no jobs, all that… It’s a big difference between then and now, like with what feels possible.

As Morris (2018: NP) notes of the multi-generational traumas of deindustrialisation, “buildings as well as ways of city life are abandoned, repurposed, made invisible”, but this does not occur “without parallel experiences at the level of the person—the socially embedded individual”.

Despite the participants not having their own memories of, or, in some cases, comprehensive knowledge about, Malden’s industrial past, they were affected by an unconscious knowledge that is “ghosted” (Bright 2011). As the participants struggled in their transitions to adulthood in contemporary Malden, some connected their experiences to the economic decline of the town since the 1980s. A collective, classed memory of struggle and a sedimented sense of oppression and marginalisation figured strongly in some of the participant’s narratives.

Things are already harder for young people here. Malden is a small place that people kind of drive through and don’t care about… Everyone here is working-class. There’s not been proper jobs here for years… Most of the young people have to leave here if they want anything above a job in a shop or café and I feel like even then the odds are against you with your background… I just feel like the options for the future are really low for young people here. (Natalie, 20)

While youth have often been overlooked in relation to deindustrialisation beyond its economic impacts, these findings support Linkon’s (2013: 39) assertion that contemporary young people occupy an important “middle space” in their inheritance of these affectively charged spaces, and “we must take their representations seriously, not for what they show us about the past but for what they reveal about what the past means in the present”. The participants in this study were living in a town that has struggled to recover from the injuries of deindustrialisation. Their challenges in finding secure employment in this context and, by extension, to take steps forwards in other aspects of their lives, seemed to be experienced as a haunting in which the young people inhabited an “unhomely present” (Morris 2018: NP). Most of the participants did not yearn for jobs in factories or mines but craved the security and “respectability” of the jobs that employed generations of people in Malden and that had enabled them to build lives and futures that resonated with their spatialised and classed identities. In the absence of desirable or resemblant alternatives in the contemporary present, ghosts of class and place surfaced, manifesting in a sense of misrecognition and injustice, and a creeping sense of dread about futures that not only seemed to divert radically from their expectations and the experiences of previous generations, but felt worryingly unpredictable.

## Haunting and (In)Justice

Haunting should not be understood as a passive state of feeling but as a prompt towards awareness and action. Gordon (2008: xvi) states that ghosts are most affectively present “when the trouble they represent… is no longer being contained or repressed or blocked from view”. These ghosts, Gordon continues, are “pregnant with unfulfilled possibility, with the something to be done that the wavering past is demanding” (p. 183). As such, haunting can be understood as inculcating understandings of the past (and present) that transcend “cold knowledge” and instead involve “transformative recognition”, where those who are haunted are invited to “reimagine social life out of a concern for justice” (Gordon 2008; Simpson 2021: NP).

Many of the participants felt that they were experiencing intergenerational injustice in their transitions to adulthood. Gordon’s (2008) “transformative recognition” was evident in many of their narratives, where “ghosts” had broken free from their moorings in the past, the trouble they represent no longer containable or deniable. The myriad challenges confronting contemporary generations of youth have not emerged suddenly. The disastrous consequences of government inaction on climate change have become increasingly obvious and catastrophic over a long period of time, while policy agendas have either exacerbated or engineered rather than ameliorated the challenges young people are encountering amid weakening opportunity structures, rising living costs, and declining state support (Pimlott-Wilson 2017; Ahlberg et al. 2021; McPherson 2024). Contemporary young people—and particularly those who are socioeconomically disadvantaged—continue to be chastised for their “low aspirations” and encouraged to be enterprising and flexible by governments, with little recognition that they are being asked to imagine and pursue futures in the context of multiple intersecting crises and with significantly less support and opportunities than previous generations (Kelly et al. 2024). Grand narratives about life patterns and temporality are collapsing, including notions of inevitable improvements in living standards in the future and, more shockingly, assumptions of continuity between the present and the future that underpin and shape much of life itself. Young people’s lives are powerfully directed on the basis of these assumptions, and they stand to lose the most from their collapse. In this context of temporal disjuncture and precarisation, the participants were haunted in ways that not only caused a longing towards seemingly “simpler times” in the past, or that created anxieties about cancelled futures, but also in ways that seemed to provoke a strong sense of injustice.

Some participants not only made sense of their lives in the present by looking to the past as an example of what was and what might have been, but also what *should* have been. Interestingly, in these parts of their narratives, the participants often shifted from first person into third person, speaking, as it were, from the vantage point of a generation of young people experiencing significantly, and unfairly, different youth transitions than previous generations.

Something has gone very wrong somewhere along the line and it’s young people who are getting shafted. (Joanna, 29)

There has to investment in places like Malden so that young people can stay and build good lives there… Our parents and their parents were able to do that, and we need to be given that. (Steven, 25)

As alluded to by Steven in the above excerpt, there was a strong sense of injustice among the participants that they were often recommended to leave their hometown for better opportunities. Three participants explicitly discussed their refusal to leave Malden as a political stance against the neoliberal expectation for young people in rural areas to relocate to urban cities. Like the majority of the participants, Finlay, 27, had deep familial roots in Malden. He spoke passionately about the expectation for young people to accept what he perceived to be substandard employment or to leave the area. He had been sanctioned by the Jobcentre for refusing to take up insecure work, citing his significant work experience and post-secondary qualifications.

I’ve taken a stand on it because it’s outrageous… Don’t get me wrong, I’ve done my share of jobs, I’ve worked in shops and McDonald’s, but I’m 27 years old, I’ve got qualifications, why should I accept that this is all there is for me?... I’m Malden born and bred and it’s when you see what they’re trying to do the place, ripping the identity and history out of it, trying to get the young people out, it’s like nah, not doing it… I should be able to have a life here.

Research suggests that young people are increasingly expressing a sense of intergenerational injustice about the gulf between their experiences and prospects and those of previous generations (e.g. Pickard 2019). Hauntology represents a valuable lens through which to not only identify the injustices that “ghosts” represent, but also to generate rich insights into how young people recognise, make sense of and respond to these injustices.

## Conclusion

By applying the concept of hauntology to the narratives of young people as they reflect on their transitions to adulthood, this paper has disrupted the futurist emphasis that encircles youth in both policy and research frames. In doing so, this paper provides fresh insights into how young people’s lives and transitions are perceived and experienced by young people in ways that evoke the past, present *and* future. This emerged in several ways in the data, including in how the participants seemed to draw on the biographies of older family members and their community to frame these aspirations as expectations for their lives, using the past as both evidence and orientation for navigating and anticipating their futures. This temporal confluence was also evident in some of the participants’ narratives about life in their hometown, where they seemed to be powerfully affected by deindustrialisation and the injuries it had cast on Malden and their classed and spatialised identities. The participants drew comparisons between the nature of employment available to previous generations with their own, expressing a strong sense of both anxiety about the future and injustice about the intergenerational discrepancies they perceived in living standards and prospects.

It has been widely theorised that the challenging socio-structural conditions of the contemporary period have left young people “trapped” in the present, unable to think or progress towards the future (Bone 2019). The findings from this study suggest that it has also led young people backwards, to reflect on how they feel life used to be when young people were able to more securely establish themselves in young adulthood. In the participants’ struggles to find secure employment or make other important life transitions, the past seemed to act as a repository of empirical evidence of what was once possible, most often what was once possible for people *like them* (i.e. their older family members in Malden), offering some a sense of hope and orientation, and others a growing sense of intergenerational injustice. As Pickering and Keightley (2006: 921) put it, looking to the past “can be both melancholic and utopian”. What is clear is that the past loomed large in most of the participants’ narratives about their transitions to adulthood in the contemporary context. It was entangled with their experiences in the present and their dispositions towards the future. The future, too, was an equally affective presence in the present; a source of anxiety, disappointment and confusion in how imperceptible and out of alignment with their expectations it appeared to be.

Scholars have argued that haunting functions to deliver a message from the past that something is derailing, suggesting danger or loss in the future with the purpose of this being made known (Jameson 1991; Gordon 2008). Many of the participants in this study seemed to be haunted by this sense of loss from both the past and the future. As they moved through their twenties without experiencing any of the historically conventional markers of adulthood, the futures they had been conditioned to expect failing to materialise or seeming remotely attainable, the past surfaced powerfully in the present as the participants tried to make sense of and respond to the temporal and structural uncertainty underpinning their transitions. These transitions were occurring against an affective backdrop charged with an eerie sense of missed final chances and a stultifying lack of viable routes through the trouble the ghosts represented (Gumbrecht 2012). This backdrop is an amplification of the eerie atmospheres described by both Derrida (1994) and Fisher (2014) in their respective hauntologies, with the very notion of continual progress that drives life under modernity being confronted by the *impossibility* of progress in a contemporary period reckoning with ecological collapse, ingrained economic uncertainty and inequality, political instability, war, and the continual erosion of identifiable, navigable paths towards stable futures (Ahlberg et al. 2021; McPherson 2024).

It is important to note that not all participants were wholly negative or anxious about their futures. The younger participants were more hopeful that things would ultimately work out, a finding that is consistent with most research with young people, which has uncovered resiliently optimistic attitudes among young people towards the future, even in the face of challenges (e.g. Keating and Melis 2021). It is also possible that this study’s interest in perceptions and experiences of social justice may have influenced some of the young people to reflect more critically on their circumstances than a study without such a theoretical framing. That said, the majority of the participants in this study did seem powerfully haunted by both the past and the future in the present in ways that track with the theorising of both Derrida and Fisher. As contemporary generations of youth face extensive challenges in their lives and future, it is important that the temporal emphasis on the future that has long surrounded young people be tempered with a meaningful recognition of temporal confluence.

## Data Availability

Data supporting this study cannot be made available because participants did not consent to their data being shared.

## Footnotes

1 “NEET” is a problematic policy label that is crudely but widely applied to a diverse group of young people who are not in education, employment or training (see Nudzor 2010).
